# Improving Crystallization and Stability of Perovskite Solar Cells Using a Low-Temperature Treated A-Site Cation Solution in the Sequential Deposition

**DOI:** 10.3390/molecules28104103

**Published:** 2023-05-15

**Authors:** Tinghao Li, Qiu Xiong, Chongzhu Hu, Can Wang, Ni Zhang, Shui-Yang Lien, Peng Gao

**Affiliations:** 1 CAS Key Laboratory of Design and Assembly of Functional Nanostructures, and Fujian Provincial Key Laboratory of Nanomaterials Fujian Institute of Research on the Structure of Matter, Chinese Academy of Sciences, Fuzhou 350002, China; 2 College of Chemistry and Materials Science, Fujian Normal University, Fuzhou 350007, China; 3 Laboratory for Advanced Functional Materials, Xiamen Institute of Rare Earth Materials, Haixi Institute, Chinese Academy of Sciences, Xiamen 361021, China; 4 University of Chinese Academy of Sciences, Beijing 100049, China; 5 School of Opto-Electronic and Communication Engineering, Xiamen University of Technology, Xiamen 361024, China

**Keywords:** low-temperature treatment, interdiffusion, crystallization, high-performance, long-term stability, two-step method, perovskite solar cell

## Abstract

The two-step sequential deposition is a commonly used method by researchers for fabricating perovskite solar cells (PSCs) due to its reproducibility and tolerant preparation conditions. However, the less-than-favorable diffusive processes in the preparation process often result in subpar crystalline quality in the perovskite films. In this study, we employed a simple strategy to regulate the crystallization process by lowering the temperature of the organic-cation precursor solutions. By doing so, we minimized interdiffusion processes between the organic cations and pre-deposited lead iodide (PbI_2_) film under poor crystallization conditions. This allowed for a homogenous perovskite film with improved crystalline orientation when transferred to appropriate environmental conditions for annealing. As a result, a boosted power conversion efficiency (PCE) was achieved in PSCs tested for 0.1 cm^2^ and 1 cm^2^, with the former exhibiting a PCE of 24.10% and the latter of 21.56%, compared to control PSCs, which showed a PCE of 22.65% and 20.69%, respectively. Additionally, the strategy increased device stability, with the cells holding 95.8% and 89.4% of the initial efficiency even after 7000 h of aging under nitrogen or 20–30% relative humidity and 25 °C. This study highlights a promising low-temperature-treated (LT-treated) strategy compatible with other PSCs fabrication techniques, adding a new possibility for temperature regulation during crystallization.

## 1. Introduction

The power conversion efficiency (PCE) of organic-inorganic metal halide perovskite solar cells (PSCs) has significantly increased over the past decade, from a mere 3.8% to an impressive 25.8%, due to the appropriate band gap and light absorption coefficient of the perovskite light absorber layer, as evidenced by numerous studies [1,2,3,4,5,6]. It has found application in photovoltaics (PVs), light-emitting diodes (LEDs), and opto-electronic studies [7,8,9]. The preparation of perovskite absorber layers usually involves either a one-step antisolvent method or a two-step sequential deposition method [10,11,12,13,14,15,16,17,18,19]. While the one-step method is simple and efficient, the two-step method, with its superior reproducibility and more tolerable preparation conditions, remains a viable alternative with an outstanding PCE rating of 25.6%, almost equal to the highest value of the one-step method. However, the complex preparation process of the two-step method presents difficulties in controlling the crystallization processes and morphology of the perovskite films. Therefore, various strategies have been proposed to address these issues, such as modifying the precursor solution, introducing additives or surfactants, and pursuing the optimal solvent [4]. With the further optimization of these methods, PCE improvements are expected for PSCs.

The two-step method involves depositing two distinct precursor layers onto a substrate in a sequential manner, which subsequently reacts to form a solid perovskite film. The precursors can exist in the form of solids, liquids, or vapors [20,21,22]. Compared to the one-step method, the two-step method presents significant challenges in achieving high-quality perovskite films. These challenges arise due to the need to control the crystallization of the PbI_2_ substrate and the organization of the organic-cation layer. These factors lead to instability issues during the ordering and crystallization of perovskite films, resulting in demanding process requirements [23,24,25,26,27]. Prior research indicates that adjusting the porosity and crystalline quality of the PbI_2_ can impact the growth process of the perovskite phase, thus enabling the production of high-quality perovskite films [26,27,28,29,30]. In the case of pre-deposited PbI_2_ films, many of the crystallization modulation strategies used in the one-step method can still be utilized because they possess comparable thermodynamic processes during crystallization. For instance, Fang, et al. integrated pentafluoroanilinium trifluoromethanesulfonate (PFAT) into PbI_2_ to manage the nucleation and crystallization of the PbI_2_ films. This technique resulted in a reduction of the Gibbs free energy of PbI_2_ nucleation and encouraged perovskite formation. Employing PbI_2_ films as porous templates facilitated the production of high-quality perovskite films with improved grain size, orientation selectivity, defect curtailment, and removal of PbI_2_ residues [31]. This significant improvement in the quality of perovskite microcrystals reduced the undesired generation of trap defects, ultimately enhancing device efficiency to 24.52% from 22.42%.

The magnification of the working area of PSCs is a potential bottleneck when using additives that may not be compatible with different precursor systems. Therefore, a simple and compatible method is required to regulate perovskite nucleation and crystal growth. The crystallization of nanoparticles in a solution is governed by nucleation and crystal growth, which can be influenced by factors such as composition, concentration, interactions, and temperature [32,33,34,35]. One-step methods benefit from low-temperature treatments as a physical approach [36,37,38,39,40]. A straightforward cold anti-solvent bath technique was proposed by Moon et al., which delays nucleation kinetics by conducting a cold anti-solvent bath at 0 °C. This technique precisely regulates the preferred orientation of perovskite crystals with large grain sizes, resulting in uniform large-area perovskite solar cells [37]. Han, et al. demonstrated that employing a cold precursor solution elevated the critical Gibbs free energy that reduced nucleation, decreased nucleation rate, and improved the overall quality of perovskite films. This enhancement led to the growth of films with smooth morphology, micron-sized grains, and preferred orientation [36]. However, unlike the two-step method, in which the PbI_2_ precursor solution did not require antisolvent and needed to be moderately warmed, regulating the film crystallization temperature through the precursor or/and antisolvent appears unfeasible in the one-step method. This is due to the distinctive preparation process employed in the two-step method that allows the regulation of perovskite crystallization through low-temperature treatment of organic-cation precursor solutions. Although this offers a unique and engaging approach to perovskite crystallization regulation, little attention has been paid, unfortunately, by researchers.

Drawing from the aforementioned factors, we successfully impeded the interdiffusion process between organic cations and PbI_2_ films, even under suboptimal crystallization conditions, by implementing a low-temperature treatment on the second-step deposited organic-cations precursor solution. This measure allowed the perovskite films to be effectively transferred to a conducive environment for annealing. Consequently, this action facilitated the preferential growth of perovskite crystals in the (100) direction, notably increasing grain size while enhancing the proportion of perovskite phases. Additionally, achieving a smoother film morphology considerably improved the transport and extraction of photogenerated carriers. In our low-temperature treated (LT-treated) two-step method for fabricating perovskite solar cells, we recorded a PCE of 24.10% and a significantly improved fill factor (FF) of 0.838 relatives to the control device with a PCE of 22.60% and FF of 0.806. Moreover, LT-treated devices demonstrated impressive stability, maintaining their initial PCE of 95.8% and 85.4% for nitrogen glove boxes and controlled humidity (20–30%), respectively, after 7000 h of storage under varying conditions. Conversely, control devices deteriorated by over 20% and 50%. This study presents an effective and straightforward approach to regulating the growth of perovskite crystals in a two-step method.

## 2. Results and Discussion

### 2.1. Mechanisms of LT Treatment

**Figure 1:**
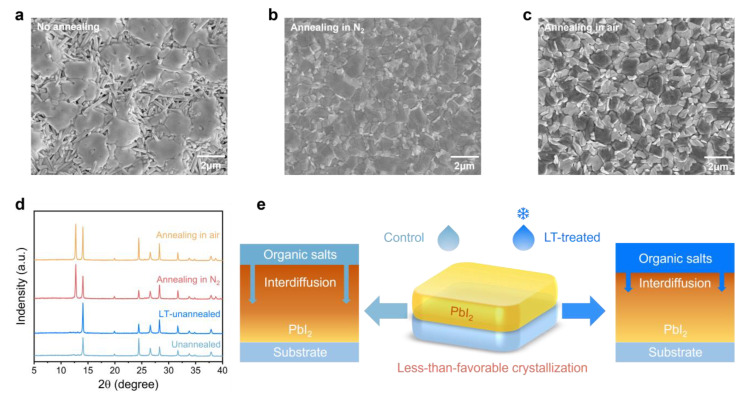
Top-view SEM images of (**a**) as-deposited, (**b**) annealing in N_2_, and (**c**) annealing in air perovskite films, respectively. The scale bar is 2 µm. (**d**) XRD patterns of various perovskite films: unannealed, LT-unannealed, annealing in N_2_, and annealing in air. (**e**) Schematic illustration (right side) of the mechanisms by which LT treatment retards the interdiffusion of organic cations and PbI_2_ under less-than-favorable crystallization conditions.

The two-step method for producing perovskite films has been shown to require annealing in a moderately humid atmosphere [41]. High temperatures during this process facilitate interdiffusion between the pre-deposited PbI_2_ and organic-cation layers. This results in grain boundary creep, which merges surrounding grains together, ultimately leading to an increase in grain size [42,43,44]. However, the deposition and annealing stages of perovskite films are discontinuous, which often leads to poor crystalline quality during the initial stages of formation due to slow interdiffusion under unfavorable crystalline conditions, such as at room temperature and under a nitrogen atmosphere. In Figure 1a, the surface scanning electron microscope morphology illustrates large lamellar perovskite grains that are still formed in the absence of thermal annealing, and this is attributed to spontaneous interdiffusion. The formation of large perovskite phases in a film is remarkable; however, they are typically present in flakes in the upper layers of the film. The lack of thermal annealing drive poses challenges for the organic-cation layer to cross the initial perovskite phase and get to the PbI_2_ layer near the buried interface, which hinders the formation of the perovskite phase. This limitation also results in the formation of numerous holes where only a part of the perovskite phase is formed, leaving the rest of the needle-like PbI_2_ or other intermediate perovskite materials unreacted. Consequently, unavoidable contact between the hole and electron transport layers occurs, leading to a short circuit within the device [45]. Furthermore, less-than-optimal interdiffusion at room temperature usually leads to non-uniform and sparse perovskite morphology.

In this study, we investigated the morphology of perovskite films under various atmospheres. Thermal annealing was found to promote the rapid formation of phase-pure perovskite resulting in flat and dense films, as illustrated in Figure 1b. In contrast, devices annealed in moist air (Figure 1c) showed uniform perovskite grains due to the moisture-dependent recrystallization process [45]. By comparison, perovskite films annealed under a nitrogen atmosphere exhibited uneven grain sizes, primarily caused by the lack of moisture. X-ray diffraction analysis confirmed the morphology observations, as displayed in Figure 1d. The full width at half-maximum (FWHM) of the (100) lattice plane, which corresponds to the perovskite crystal located at 14.06°, decreased from 0.160° to 0.126° for the nitrogen annealed films, indicating an increased average grain size. Additionally, the peak intensity of the (111) lattice plane at 24.44° was notably higher compared to the other samples, indicating a significant enhancement in the crystallinity of the perovskite film.

Therefore, to achieve high-quality perovskite films, it is crucial to suppress the interdiffusion of functional layers under unfavorable crystallization conditions. Thus, a low-temperature treatment was employed to cool down the organic-cation precursor solution. The interdiffusion coefficient between the functional layers can be calculated using classical diffusion kinetics with the following Equation (1):

(1) $$D\;=D_{0}\exp\left(-\frac{Q}{\mathrm{RT}}\right),$$

where D is the diffusion coefficient, D_0_ is the diffusion factor, Q is the diffusion activation energy, and R is the gas constant [46]. It is observed that the diffusion rate increases with an increase in diffusion coefficient, which is positively correlated with temperature. Molecular dynamics can explain this phenomenon since, with an increase in temperature, the average kinetic energy of the molecules enhances, making it more likely for them to cross the diffusion activation energy barrier, thus increasing the diffusion rate. For simplicity, in the following study, untreated and LT-treated devices are referred to as “control” and “LT-treated”, respectively. As depicted in Figure 1e, it can be deduced that the LT-treated organic-cation solution reduces the diffusion rate of PbI_2_, with the organic-cation layer, in a less-than-favorable crystallization environment. This process slows down the diffusion as much as possible to ensure that the crystallization process occurs under optimal environmental conditions.

The results presented in Figure 1d reveal that the LT-treated perovskite films exhibit superior crystallinity and crystallographic orientation compared to the untreated films, thereby supporting the aforementioned hypothesis. Furthermore, the absence of distinct diffraction peaks of PbI_2_ in the unannealed films implies that the annealing process results in the decomposition of some of the perovskite, leaving behind only the remaining PbI_2_ [45]. As shown in Figure S1, we also observed the change in the external appearance of the perovskite films treated by different conditions but without annealing. Specifically, it is noticeable that the untreated films transition to the black perovskite phase within 10 min. Meanwhile, the LT-treated films exhibit a slower transformation, thereby demonstrating their superior resistance to unfavorable crystallization environments.

### 2.2. Film Characterization

**Figure 2:**
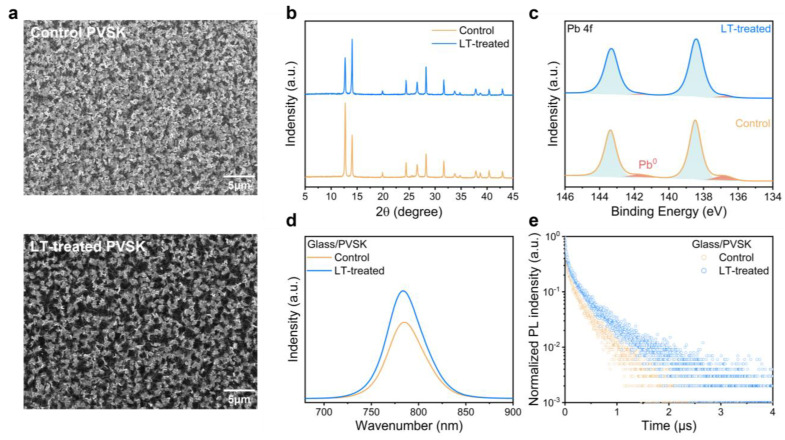
(**a**) Top-view SEM images, (**b**) XRD patterns, and (**c**) XPS spectra of Pb 4f of perovskite film with and without LT-treated. (**d**) ss-PL spectra and (**e**) tr-PL spectra of perovskite with and without LT-treated coated on glass.

Further, we conducted an optical analysis of differently treated perovskite films. SEM imaging showed the surface morphology of perovskite films, as depicted in Figure 2a. As observed, the LT-treated film had larger, more abundant black perovskite grains, while the control film had primarily white PbI_2_ grains. Moderate amounts of PbI_2_ can effectively passivate traps, whereas excessive amounts can negatively affect device stability [10,11,17]. The diffusion crystallization process executed under more favorable environmental conditions could be responsible for the larger grain formation in the LT-treated samples. Additionally, the roughness analysis of the atomic force microscopy (AFM) image in Figure S2 illustrated that the LT-treated film had a slightly lower roughness value of 19.33 nm than the control film, with a value of 22.39 nm. Reduced roughness facilitated interfacial contact, effectively reducing carrier transport losses between the hole transport layer and the perovskite layer.

We further analyzed the X-ray diffraction (XRD) patterns of two perovskite films, as presented in Figure 2b, and identified a diffraction peak at 12.7°, corresponding to the (001) lattice plane of hexagonal PbI_2_. The decrease in the diffraction intensity of PbI_2_ and the corresponding increase in perovskite intensity indicates a more substantial reaction between the organic cations and PbI_2_, resulting in the formation of perovskite in the film. To determine the grain size of perovskite crystals, we used Scherrer’s formula, which involved examining the FWHM of the (100) reflection of the perovskite crystal at 14.06°. We found that the FWHM value decreased from 0.161° to 0.135° in the LT-treated film compared to the control film. This decrease suggested an improvement in the perovskite grain size, which is consistent with the SEM findings. 

The X-ray photoelectron spectroscopy (XPS) images of both perovskite films are presented in Figure 2c and Figure S3. The two shoulder peaks situated adjacent to the primary Pb 4f peaks represent Pb^0^, which typically appear due to deep-level traps induced by residual PbI_2_ in the perovskite film following exposure to light or radiation. These traps are known to cause undesirable carrier separation and transport behaviors [47]. Interestingly, the LT-treated film exhibited relatively fewer Pb^0^ compared to the control film. Moreover, the I/Pb ratio of the treated and control films was compared, as shown in Figure S4, using data extracted from the XPS patterns. It was observed that the ratio increased to 2.67 after treatment, resembling the ideal value of 3. This increase confirms the strong ability of LT-treated organic cations to effectively bind with PbI_2_, which supports the results obtained through SEM and XRD. Furthermore, the higher percentage of perovskite crystals signifies an improved potential for light absorption, whereas the increased percentage of PbI_2_ implies a higher probability of decomposition, leading to deep energy level trap production.

We evaluated the Ultraviolet-visible (UV-vis) absorption measurements of both perovskite films (Figure S5) and found no significant changes in their absorption across different wavelengths. Next, we employed steady-state photoluminescence (ss-PL) and time-resolved photoluminescence (tr-PL) spectroscopy to analyze the non-radiative combination processes within the films deposited on glass substrates. Our observations of the films’ absorption wavelengths (Figure 2d) indicate that the band gap was also unaffected, corroborating the results from UV-vis spectrometry. Nevertheless, we noted a rise in the excitation intensity of the LT-treated film relative to the control film. This observation suggests that fewer carriers are trapped by traps, thereby showing a lower non-radiative combination process, resulting in reduced energy loss during carrier separation and transport. This finding can be explained by the high crystalline quality perovskite films formed via LT treatment, resulting in fewer internal traps in the film. Lastly, we evaluated the lifetime of photogenerated electrons and holes in both perovskite films. The tr-PL intensity decay spectra were fitted with the following bi-exponential decay Equation (2):

(2) $$I\left(t\right)={\;\gamma }_{0}+A_{1}\exp\left(-\frac{t}{\tau_{1}}\right)+A_{2}\exp\left(-\frac{t}{\tau_{2}}\right),$$

where γ_0_ is the decay constant, A_1_ and A_2_ are decay amplitudes, τ_1_ is the fast interface charge extraction, and τ_2_ is the slow trap-assisted recombination [48]. As shown in Figure 2e and Table S1, our observations demonstrate a significant enhancement in the fitted lifetime from 199 ns to 283 ns of the LT-treated film when compared to the control device. This finding is notable as it suggests that the LT treatment has extended the photogenerated electron-hole pair lifetime within the perovskite film. An extended electron-hole pair lifetime is a crucial factor linked to extended exciton diffusion length. Furthermore, the LT-treated film exhibited improved charge transport properties, which promotes an efficient charge extraction mechanism, ultimately leading to superior device performance.

### 2.3. Carrier Dynamic Characterization

**Figure 3:**
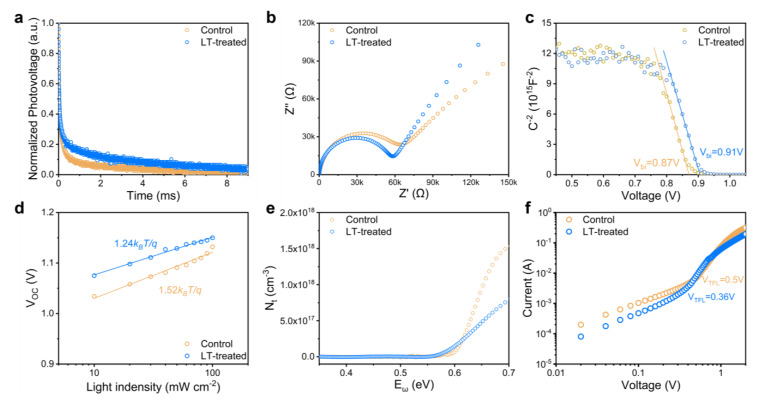
(**a**) Transient photovoltage (TPV) decay, (**b**) The Nyquist plots, (**c**) Mott-Schottky plots, (**d**) Relationship between V_OC_ and the light intensity, (**e**) Calculated trap density (N_t_), and (**f**) Dark J-V curves of electron-only devices for PSCs with and without LT-treated. The empty circles indicate the measured data, and the solid lines indicate the fitted curves in (**c**,**d**).

Electrochemical characterization techniques were employed to gain insight into the carrier characteristics and trap density in PSCs. Specifically, we utilized transient photovoltage (TPV) decay measurements, as shown in Figure 3a, to investigate the carrier recombination behavior within PSCs. To determine the time constant for charge recombination (τ_R_), we fitted the TPV data with a single exponential decay model. Our analysis revealed that the τ_R_ values for both the control and LT-treated devices were 0.18 ms and 1.12 ms, respectively, indicating that the carrier recombination process was significantly hindered in the LT-treated device compared to the control device. Moreover, the improved crystalline quality of the perovskite film resulting from LT treatment effectively suppressed the electron-hole pair recombination rate, consistent with the trends observed in photoluminescence (PL) intensity and lifetime measurements.

We further conducted electrochemical impedance spectroscopy (EIS) measurements to evaluate the carrier recombination and transport properties in PSCs. The Nyquist plot (Figure 3b) was analyzed to assign charge transport resistance (R_ct_) and recombination resistance (R_rec_), which were represented by two semicircles with different radii. Our observations showed a significant decrease in R_ct_ and an increase in R_rec_ for the LT-treated film compared to the control device, consistent with TPV test results (Table S2). LT treatment likely enhanced the perovskite film’s crystalline quality, facilitating smooth carrier transport within the layer. Improvements in surface morphology also fostered favorable interfacial contact with the hole transport layer, reducing interfacial carrier recombination and promoting efficient carrier extraction. Notably, the increase in recombination resistance was more prominent than the reduction in transmission resistance, indicating effective suppression of carrier recombination and reduced defect density within the perovskite film.

The Mott–Schottky diagram of the device is shown in Figure 3c. The built-in potentials of the control and LT-treated devices were affirmed to be 0.87 and 0.91 V, respectively, consistent with the trend of the open circuit voltage (V_OC_) measured in subsequent devices. To gain insight into the non-radiative recombination dynamics within the device, we also tested the trend of the short-circuit current density (J_SC_) at different light intensities. As shown in Figure 3d, the LT-treated device exhibits a smaller slope (1.24 k_B_t/q) than the control device (1.52 k_B_t/q), indicating less trap-assisted Shockley–Read–Hall recombination.

Thermal admittance spectroscopy (TAS) measurements were conducted to determine the trap density (N_t_) within perovskite solar cells [49,50,51]. As depicted in Figure 3e, N_t_ values showed a significant decrease for the LT-treated film compared to the control film, particularly for deep-level traps exceeding 0.6 eV. The LT-treated devices exhibited an almost twofold reduction in trap density, suggesting that the LT treatment can substantially lower the trap density inside or on the surface of the perovskite film, leading to superior device performance. In addition, we utilized the space-charge-limited-current (SCLC) technique to verify the computed trap densities of the perovskite films with FTO/SnO_2_/Perovskite/PCBM/Au structures, as displayed in Figure 3f [52]. The results of the calculations demonstrated that the electron trap density inside the LT-treated device was 1.86 × 10^15^ cm^−3^, which was significantly lower than the 3.02 × 10^15^ cm^−3^ observed in the control device.

Based on the comprehensive analysis presented above, we infer that the effective reduction of trap density within the PSCs achieved through the LT-treated strategy is primarily responsible for the improved photovoltaic performances of the devices. The LT-treated strategy enhances the crystal quality of perovskite films and reduces the trap density, thereby lowering the likelihood of photogenerated carriers being ensnared by defects. Additionally, the decreased grain boundaries minimize the obstruction of carrier transport. The favorable carrier transport properties lead to an improved charge diffusion length, enabling smoother transporter delivery to the metal electrode. These factors cumulatively enhance the charge-harvesting efficiency of PSCs, thereby enhancing the photovoltaic performance of the devices.

### 2.4. Photovoltaic Performance and Stability of PSCs

**Figure 4:**
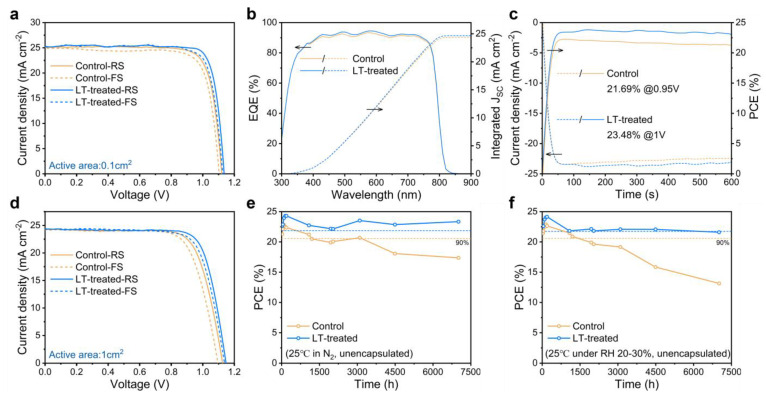
(**a**) J-V curves of the 0.1 cm^2^ PSCs. (**b**) EQE spectra and corresponding integrated J_SC_ of the PSCs. (**c**) Stabilized maximum power output at the maximum power point (MPP) to obtain the stabilized PCEs. (**d**) J-V curves of the 1 cm^2^ PSCs. (**e**,**f**) long-term stability of unencapsulated PSCs in (**e**) N_2_ glove box and (**f**) an ambient environment of 20–30% relative humidity at 25 °C.

We explored the practical effects of the LT-treated strategy on the photovoltaic performance of perovskite solar cells. Fully structured devices comprising FTO/SnO_2_/Perovskite/Spiro-OMeTAD/Au were fabricated, and the current density–voltage (J-V) curves of champion PSCs with and without LT treatment processes were compared, as depicted in Figure 4a. The corresponding photovoltaic performance parameters are detailed in Table S3. We found that the LT-treated devices exhibited significant enhancements in all photovoltaic parameters. Specifically, the FF demonstrated the most significant increase of approximately 4.1%, rising from 80.59% to 83.88%. The V_OC_ and J_SC_ also showed varying degrees of enhancement under reverse scan, with an increase from 1.121 V to 1.136 V for V_OC_ (a 1.3% enhancement) and from 25.07 mA/cm^2^ to 25.30 mA/cm^2^ for J_SC_ (a 0.9% enhancement). As a result, the champion PCE of the LT-treated devices was enhanced by 6.4%, rising from 22.65% to 24.10%. These results provide compelling evidence that the LT-treated strategy can efficiently enhance the overall device performance. Moreover, we observed even better reproducibility for LT-treated devices in the statistical data of device performance, as shown in Figure S6.

The external quantum efficiency (EQE) spectra of the corresponding devices are illustrated in Figure 4b. It can be observed that LT-treated devices exhibit an increased absorption over a broad wavelength range. The calculated integrated J_SC_ values for the control and LT-treated devices were found to be 24.35 and 24.66 mA/cm^2^, respectively, differing by less than 5% from the J_SC_ value obtained from the J-V measurement, suggesting reasonable values. Furthering our analysis in Figure 4c, we performed stable output power (SPO) measurements of the devices at a fixed bias voltage. The stable PCEs were recorded as 21.69% and 23.48% for the control and LT-treated devices, respectively, which are consistent with the PCE values obtained by the J-V measurement. To validate the effectiveness of the LT-treated strategy on a larger active area, we fabricated a 1 cm^2^ device presenting its J-V curves for champion efficiency in Figure 4d. Table S4 presents the corresponding photovoltaic performance parameters. We note that the PCE values for larger perovskite solar cells with an LT-treated strategy are marginally lower compared to those of 0.1 cm^2^ devices with reduced J_SC_ and FF values. This trend could be attributed to the increased series resistance. The V_OC_ values for the control and LT-treated devices were measured at 1.126 V and 1.147 V, respectively, while the J_SC_ values were 24.21 mA/cm^2^ and 24.33 mA/cm^2^. The FF values were 74.46% and 77.21%, respectively, as shown in Table S4.

In addition to assessing the static photovoltaic performance, we monitored the time-dependent evolution of the PCE for both the control and LT-treated PSCs under various environmental conditions. The evolution of PCE was analyzed via testing in a nitrogen glove box at room temperature, as shown in Figure 4e. Our results indicated that the PCE of the control PSCs decreased by more than 20% after aging for 7000 h, while the LT-treated PSCs maintained 95.8% of their initial PCE over the same period. To investigate the humidity stability of the PSCs, we conducted tests by storing the unencapsulated devices in a dry cabinet at room temperature with controlled humidity levels (20–30%). As depicted in Figure 4f, the LT-treated PSCs retained 89.4% of their initial PCE, whereas the control PSCs lost nearly 50% of their initial PCE after aging for 7000 h. These findings highlight the superior long-term stability of LT-treated PSCs. It is important to note that the long-term stability tests revealed considerable fluctuations in the device’s performance. The observed sudden drops in PCE might be attributed to irreversible damage from electrochemical testing. In contrast, the increase in the device’s efficiency could be linked to the gradual maturation of PSCs during storage and the full oxidation of the hole transport layer upon exposure to air before testing.

## 3. Materials and Methods

### 3.1. Reagents and Materials

SnO_2_ colloid precursor (tin (IV) oxide, 15% in H_2_O colloidal dispersion) was obtained from Alfa Aesar, Haverhill, MA, USA. Lead iodide (PbI_2_, 99.99%) was acquired from TCI. Formamidinium iodide (FAI), methylammonium bromide (MABr), and methylammonium chloride (MACl) were synthesized by us. The 2,2′,7,7′-tetrakis [N, N-di(4-methoxyphenyl) amino]-9,9′-spirobifluorene (Spiro-OMeTAD) was bought from Shenzhen Feiming Co., Ltd., Shenzhen, China. Anhydrous N, N-dimethylformamide (DMF), dimethyl sulfoxide (DMSO), isopropanol (IPA), chlorobenzene (CB), and acetonitrile were obtained from Sigma-Aldrich. Bis(trifluoromethane)sulfonimide lithium salt (Li-TFSID, 99.95%) and 4-tert-butylpyridine (t-BP, 98%) were also procured from Sigma-Aldrich. The Fluorine-doped SnO_2_-coated glass slide (FTO) was bought from Advanced Election Technology Co., Ltd., Riyadh, Saudi Arabia.

### 3.2. Preparation of Precursor Solutions

In accordance with standard protocols for the preparation of perovskite solar cells, all precursor solutions were prepared under stringent conditions, including the use of an N_2_ glovebox, high-purity solvents, precise pipettes, and balances. Notably, the SnO_2_ solution was an exception because it was prepared in air. The synthesis of the perovskite precursor solution comprised two essential steps. First, a PbI_2_ precursor solution was generated by dissolving 1.45 M PbI_2_ in DMF and anhydrous DMSO in a volume ratio of 9:1. Next, an organic amine salt solution was created by dissolving 70 mg FAI, 7 mg MABr, and 7 mg MACl in 1 mL of IPA. Following this, the Spiro-OMeTAD solution was generated by dissolving 72.3 mg of Spiro-OMeTAD in 1 mL of anhydrous chlorobenzene. Upon complete dissolution of Spiro-OMeTAD, 17.5 µL of Li-TFSI solution (520 mg mL^−1^ in acetonitrile) and 28.8 µL of t-BP were added. All prepared solutions were allowed to be stirred overnight and subsequently filtered before use to ensure optimal purity and homogeneity.

### 3.3. Fabrication of the Devices

The perovskite solar cells were fabricated on fluorine-doped tin oxide (FTO, NSG) substrates with a 6 mm blank area etched on top of each substrate. The FTO substrates were meticulously cleaned using a multistep procedure. First, they were subjected to a cleaning process involving detergent, followed by sequential ultrasonic cleaning in deionized water, acetone, and ethanol, with a duration of 15 min for each solvent. Subsequently, the substrates were dried in a cabinet at 70 °C for 30 min to eliminate residual solvent. Prior to the deposition of SnO_2_ and the perovskite layer, the substrates were cleaned using plasma treatment for 10 min. For the deposition of the SnO_2_ film, 80 μL of the SnO_2_ colloid solution was spin-coated onto the cleaned FTO substrates at 3000 rpm for 30 s, then annealed at 150 °C for 30 min. For the deposition of the perovskite layer, a 45 μL PbI_2_ solution was spin-coated onto the SnO_2_ substrate in the N_2_ glove box at 1500 rpm for 30 s and annealed at 70 °C for 1 min. Upon cooling the PbI_2_ solution to room temperature, a 50 μL organic amine salt solution was spin-coated onto the PbI_2_ film at 1300 rpm for 30 s. The coated film was subsequently removed from the N_2_ glove box and annealed in ambient air (with relative humidity ranging between 30% to 40%) at 150 °C for 15 min.

It should be noted that the organic ammonium salt solution should always be placed in a refrigerator at 0 °C to keep it at a low temperature during the spin coating process for LT-treated devices. Therefore, before spin-coating the PbI_2_, organic amine salt, and Spiro-OMeTAD solutions, the nitrogen glove box was re-flushed for several minutes to eliminate any residual solvent vapors from the previous steps. The hole transport layer was then deposited on top of the perovskite layer via 4000 rpm spin-coating of the Spiro-OMeTAD solution after allowing the perovskite layer to cool. Finally, an 80 nm gold electrode was thermally evaporated onto the back contact using a shadow mask under 5 × 10^−5^ Pa and at a thermal evaporation rate of 0.05–0.5 Å s^−1^. Two device sizes were utilized: small device areas of 0.1 cm^2^ and large device areas of 1 cm^2^.

### 3.4. Characterization

Scanning electron microscope (SEM) images were observed by field emission electron microscopy (Apreo S, Thermo Scientific™, Franklin, MA, USA). X-ray diffraction (XRD) patterns were measured by Rigaku X-ray diffractometer (Cu Kα radiation, λ = 1.5418 Å). Atomic force microscopy (AFM) measurements were conducted using a Bruker Multimode-8J (Bruker, Billerica, MA, USA) microscope ScanAsyst air mode. An X-ray photoelectron spectroscopy system (Axis Supra, Shimadzu, Kratos, Manchester, UK) was used to obtain X-ray photoelectron spectra (XPS), using Al Kα X-ray radiation (1486.6 eV) as the X-ray source. Absorption spectra were analyzed by a UV-vis spectrophotometer (Cary 5000, Agilent, Santa Clara, CA, USA). Photoluminescence (PL) and time-resolved photoluminescence (TRPL) experiments were conducted using a series of Fluorescence spectrometers (FLS-980, Edinburgh Instruments, Edinburgh, Livingston, UK), with the excitation and emission wavelengths for TRPL measurement being 480 and 790 nm, respectively. The transient photovoltage (TPV, Electrochemical impedance spectroscopy (EIS), thermal admittance spectroscopy (TAS), and Mott–Schottky measurements were carried out using an electrochemical workstation (Zennium Zahner, Kronach, Germany). The photovoltaic performance was evaluated under AM 1.5 G (0.1 W/cm^2^) illumination using a solar simulator (Enli Tech, Beijing, China) and measured on a Keithley 2401 source meter. A source meter (Keithley 2401, Keithley, China) was also employed to record current density-voltage (J-V) characteristics with a scan speed of 100 mV/s. External quantum efficiency (EQE) spectra were acquired at DC mode using an EQE system (QE-R 3011, Enli Tech, China). Stability tests were performed by separately storing the device samples in the air for a specific duration to allow for oxidation before performing the test. The trap density was determined from the frequency–capacitance relationship, which was modeled through the following Equation (3):

(3) $$N_{t}\left(E\omega \right)=-\frac{V_{bi}}{qW}\frac{dC}{d\omega }\frac{\omega }{k_{B}T},$$

where *V_bi_*, *W*, *C*, *ω*, *k_B_*, and *T* correspond to the built-in potential, depletion width, capacitance, angular frequency, Boltzmann constant, and temperature, respectively [51]. The corresponding energetic demarcation (*E_ω_*) could be defined with ω according to the following Equation (4):

(4) $$E_{\omega }=k_{B}Tln\left(\frac{\omega_{0}}{\omega }\right),$$

where *ω*_0_ is the attempt-to-escape frequency [49,50]. Space-charge-limited-current (SCLC) measurement was performed on a Keithley 2401 source meter ranging from 0 V to 5 V. As a result, *N_t_* can be calculated from the following Equation (5):

(5) $$N_{t}=\frac{2V_{TEL}\varepsilon_{r}\varepsilon_{0}}{qL^{2}},$$

where *V_TFL_*, *ε_r_*, *ε*_0_, *q*, and *L* correspond to the trap-filling voltage, relative dielectric constant, vacuum permittivity, elementary charge, and perovskite film thickness, respectively [52].

## 4. Conclusions

In summary, this study proposes a temperature control strategy for producing high-performance perovskite solar cells via an LT-treated strategy of organic-cations solutions that were spin-coated in the second step of the two-step method. The LT treatment of organic-cations precursor solutions significantly slowed down the interdiffusion process, even in unfavorable crystallization environments, which facilitated their transfer to more suitable environmental conditions for annealing. This resulted in the preferential growth of perovskite crystals and the enlargement of grain size with a smooth morphology, leading to the reduction of trap density inside the device and better carrier transport/extraction. The LT-treated device achieved a remarkable PCE of 24.10%, and the champion device maintained its initial efficiency of 95.8% and 89.4% for the unencapsulated device, respectively, after aging for over 7000 h in a nitrogen glove box and a dry cabinet with controlled humidity levels (20–30%). Furthermore, our research suggests that the LT-treated technique in the two-step method could be adapted to other strategies, offering a wide universal reference value and demonstrating strong feasibility.

## Data Availability

All data are available upon request.

## Supplementary Materials

The following supporting information can be downloaded at: https://www.mdpi.com/article/10.3390/molecules28104103/s1, Figure S1: Optical graphs of perovskite films after deposition of organic salts as a function of time; Figure S2: AFM images of perovskite films without (a) and with (b) LT treatment; Figure S3: The entire XPS spectra of (a) control and (b) LT-treated perovskite films; Figure S4: Radio of I/Pb of control and LT-treated perovskite films from XPS spectra; Figure S5: UV-visible absorption of control and LT-treated perovskite films; Figure S6: PCE histogram of PSCs without and with LT-treatment based on 20 individual PSCs in each batch; Table S1: The fitted lifetime and parameters deduced from the bi-exponential decay kinetic; Table S2: The fitting results of EIS spectra of PSCs; Table S3: Photovoltaic parameters of the best-performing PSCs with 0.1 cm^2^ active area; Table S4: Photovoltaic parameters of the best-performing PSCs with 1 cm^2^ active area.
