# Supplementary material for: Improving Crystallization and Stability of Perovskite Solar Cells Using a Low-Temperature Treated A-Site Cation Solution in the Sequential Deposition
Source: Molecules. 2023 May 15;28(10):4103. doi: 10.3390/molecules28104103 (PMC10223571; doi:10.3390/molecules28104103)
Supplement: Supplementary file 1 [file molecules-28-04103-s001.zip › molecules-2388501-supplementary.pdf]

Supporting Information

Improving Crystallization and Stability of Perovskite Solar Cells Using a Low-Temperature Treated A-Site Cation Solution in the Sequential Deposition

Tinghao Li [1,2,3], Qiu Xiong [1,3,4], Chongzhu Hu [1,3], Can Wang [1,3,4], Ni Zhang [1,3,4], Shui-yang Lien [5] and Peng Gao [1,3,4,*]

1 CAS Key Laboratory of Design and Assembly of Functional Nanostructures, and Fujian Provincial Key Laboratory of Nanomaterials Fujian Institute of Research on the Structure of Matter, Chinese Academy of Sciences, Fuzhou 350002, China
2 College of Chemistry and Materials Science, Fujian Normal University, Fuzhou 350007, China
3 Laboratory for Advanced Functional Materials, Xiamen Institute of Rare Earth Materials, Haixi Institute, Chinese Academy of Sciences, Xiamen 361021, China
4 University of Chinese Academy of Sciences, Beijing 100049, China
5 School of Opto-electronic and Communication Engineering, Xiamen University of Technology, Xiamen 361024, China
* Correspondence: peng.gao@fjirsm.ac.cn

Supplemental items

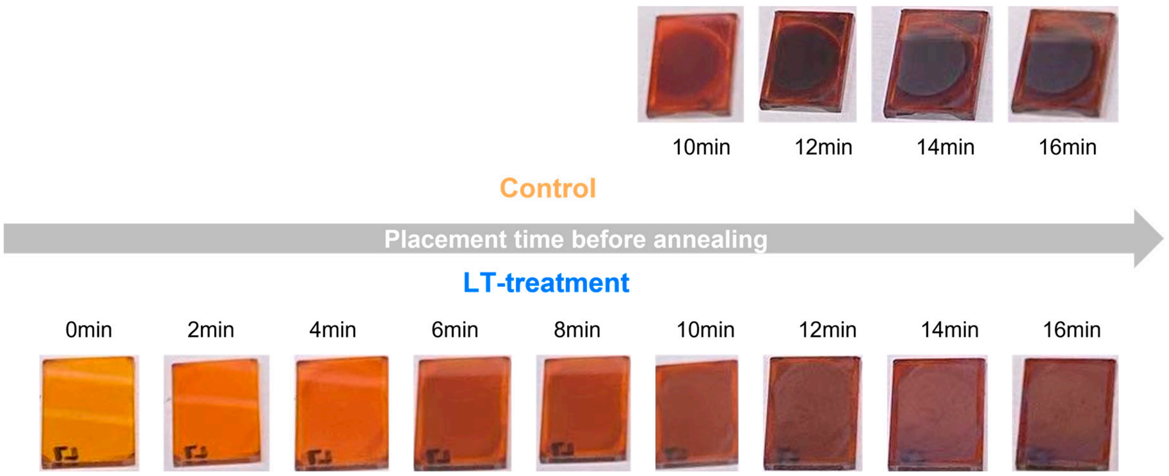

Figure S1. Optical graphs of perovskite films after deposition of organic salts as a function of time.

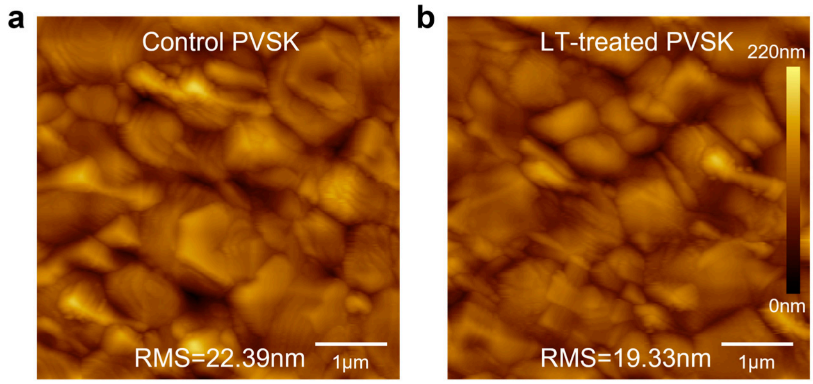

Figure S2. AFM images of perovskite films without (a) and with (b) LT treatment.

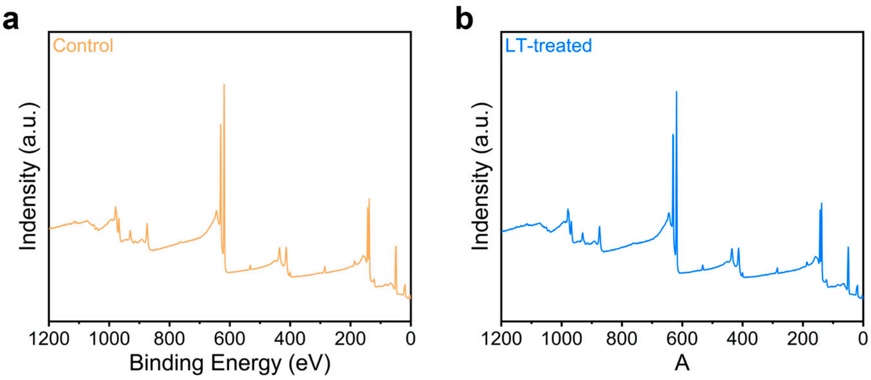

Figure S3. The entire XPS spectra of (a) control and (b) LT-treated perovskite films.

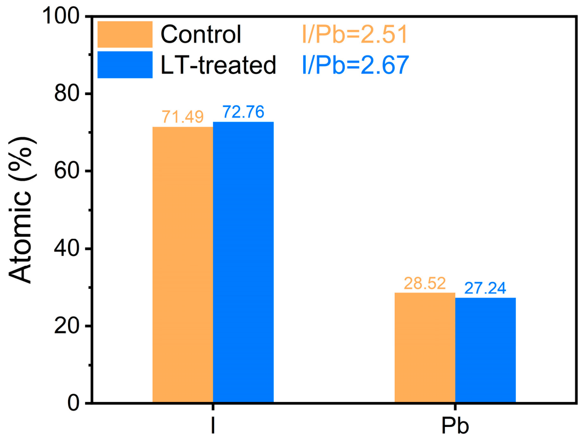

Figure S4. Radio of I/Pb of control and LT-treated perovskite films from XPS spectra.

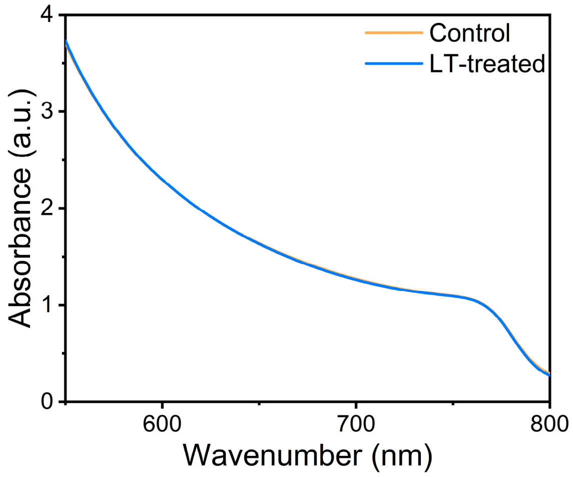

Figure S5. UV-visible absorption of control and LT-treated perovskite films.

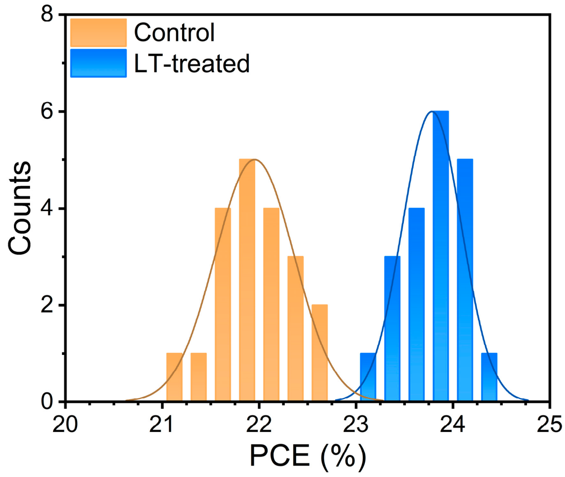

Figure S6. PCE histogram of PSCs without and with LT-treatment based on 20 individual PSCs in each batch.

Table S1. The fitted lifetime and parameters deduced from the bi-exponential decay kinetic.

Samples	τ_1 (ns)	A_1	τ_2 (ns)	A_2	τ_{AVE} (ns)
Control	19.03	0.70	238.50	0.26	199.69
LT-treated	23.50	0.64	322.70	0.31	283.88

Table S2. The fitting results of EIS spectra of PSCs.

Samples	R_s (Ω)	R_{ct} (Ω)	R_{rec} (Ω)	C (10^{-8}F)	$CPE\text{-}T$ (10^{-6}F)	$CPE\text{-}P$
Control	7.47	56217	456980	1.09	1.47	0.64
LT-treated	8.55	54514	806620	1.02	2.51	0.70

Table S3. Photovoltaic parameters of the best-performing PSCs with 0.1cm^2 active area.

Devices	V_{OC} (V)	J_{SC} (mA/cm^2)	FF (%)	PCE (%)	HI
Control RS	1.121	25.07	80.59	22.65	0.045
Control FS	1.103	24.89	78.73	21.62	
LT-treated RS	1.136	25.30	83.88	24.10	0.042
LT-treated FS	1.126	25.20	81.29	23.07	

Table S4. Photovoltaic parameters of the best-performing PSCs with 1cm^2 active area.

Devices	V_{OC} (V)	J_{SC} (mA/cm^2)	FF (%)	PCE (%)	HI
Control RS	1.126	24.21	74.46	20.29	0.043
Control FS	1.096	24.30	72.89	19.41	
LT-treated RS	1.147	24.33	77.21	21.56	0.039
LT-treated FS	1.138	24.27	74.99	20.71	
